# The gut microbiome of laboratory mice: considerations and best practices for translational research

**DOI:** 10.1007/s00335-021-09863-7

**Published:** 2021-03-10

**Authors:** Aaron C. Ericsson, Craig L. Franklin

**Affiliations:** University of Missouri Metagenomics Center (MUMC), MU Mutant Mouse Resource and Research Center (MU MMRRC), Department of Veterinary Pathobiology, College of Veterinary Medicine, University of Missouri, Columbia, MO USA

## Abstract

Just as the gut microbiota (GM) is now recognized as an integral mediator of environmental influences on human physiology, susceptibility to disease, and response to pharmacological intervention, so too does the GM of laboratory mice affect the phenotype of research using mouse models. Multiple experimental factors have been shown to affect the composition of the GM in research mice, as well as the model phenotype, suggesting that the GM represents a major component in experimental reproducibility. Moreover, several recent studies suggest that manipulation of the GM of laboratory mice can substantially improve the predictive power or translatability of data generated in mouse models to the human conditions under investigation. This review provides readers with information related to these various factors and practices, and recommendations regarding methods by which issues with poor reproducibility or translatability can be transformed into discoveries.

## Introduction

Owing to its metabolic and biotransformative capacity, the mammalian gut microbiota (GM) is now frequently regarded as a quasi-organ (Clarke et al. 2014; O’Hara and Shanahan 2006), with a collective metagenome dwarfing the host genome in terms of complexity and diversity. In addition to its profound influence on developmental processes and digestion and assimilation of nutrients, the GM also harvests carbon from xenobiotic compounds in the gut lumen, often changing the half-life and activity of parent compounds (Koppel et al. 2017), and is similarly responsible for other catabolic processes in the gut lumen. It follows that differences between individuals in the composition of their GM might, at least partially, explain differences in disease susceptibility or response to treatment (Gopalakrishnan et al. 2018; Routy et al. 2018; Matson et al. 2018). Accordingly, the biomedical research community has invested tremendous time and resources in endeavors like the Human Microbiome Project, the goal of which is to characterize the healthy human microbiota of various anatomic sites, and hundreds of other related studies examining deviations from the norm in various disease settings. Comparative studies using animal models have been critical to test the causality of associations found in human patients, and to define mechanisms underlying those associations.

Accordingly, there is a growing realization that the GM of laboratory mouse models must be considered in the context of biomedical research as a whole. For a researcher to not know the specific strain of mouse used in their experiments would be laughable, yet many researchers have minimal information regarding the GM of the mice in their research colonies and how it might be influencing the phenotype of their model. Indeed, multiple studies using mouse models have recapitulated or predicted relationships between the GM and host health in humans (Ivanov et al. 2009; Chen et al. 2018; Rosshart et al. 2019; Shin et al. 2014; Cuesta-Zuluaga et al. 2017), reflecting the utility of translational research in this field. With this in mind, the influences of the GM are implicated in three major facets of biomedical and translational research—reproducibility, translatability, and discovery. Here, we present a broad overview of these considerations, including current knowledge and best practices, with the goal of enhancing all three components.

## The GM as a central hub in model outcomes

Normal physiology and susceptibility to disease are influenced by both the host genome and environmental factors including the gut microbiome and its metagenome. Notably, the GM is extremely dynamic and is itself influenced by the host genome (Davenport et al. 2015; Org et al. 2015; Wang et al. 2016), health status (Vos and Vos 2012), and myriad external environmental factors, resulting in a complex and circular relationship. While the GM of various host species share a high degree of homology at the taxonomic level of family and even genus, the bacterial species and strains within the mammalian GM are specific to their cognate host species (Chung et al. 2012), highlighting the value of studying murine models with murine GM. In research animals and humans alike, the GM functions as both a dependent variable affected by factors leading to changes in its taxonomic composition or function, and an independent variable associated with subsequent changes in host physiology and disease phenotypes. It is therefore reasonable to consider the GM as a mechanism by which virtually any environmental factor, including psychological stress (Bailey et al. 2011), might alter experimental outcomes. A careful reading of commentaries related to the ‘Reproducibility Crisis’ (Perrin 2014; Collins and Tabak 2014; Mogil 2017; Ramirez et al. 2017) affecting biomedical research suggests that there are actually two separate crises characterized by poor experimental reproducibility within and between labs, but also poor inter-species reproducibility of scientific findings, including translatability to the human condition. While certain factors related to experimental design and statistical analysis have been identified as one source of poor reproducibility, a wealth of data, a fraction of which are presented here, suggest that the GM is a critical determinant of the reproducibility and translatability of research performed using animal models. With this in mind, several lines of research have provided meaningful insights into broadly applicable approaches and practices to enhance reproducibility between labs and improve the predictive power and translatability of mouse models to the outcomes observed in humans. Much of this research has also resulted in ground-breaking discoveries in the fields of immunology, neurology, and endocrinology, reinforcing the incredible influence of the GM on the development and function of virtually every facet of host health.

## Experimental reproducibility as a function of the GM

Regarding factors capable of influencing the GM of laboratory animals, the list continues to grow and includes source of the animals (Hirayama et al. 1990; Hufeldt et al. 2010; Ericsson et al. 2015), diet (Org et al. 2015; Ericsson et al. 2018), caging (Ericsson et al. 2018; Lundberg et al. 2017), bedding (Ericsson et al. 2018; Bidot et al. 2018), water treatment (Bidot et al. 2018), transportation (Montonye et al. 2018; Ma et al. 2012), housing density (Bogatyrev et al. 2020; Basson et al. 2020), sex (Org et al. 2016; Kozik et al. 2017), genetics (Hufeldt et al. 2010; Ericsson et al. 2015; Hildebrand et al. 2013; Kovacs et al. 2011) and a wide range of antibiotics and other pharmacological agents (Korte et al. 2020; Zhao et al. 2020; Yin et al. 2015; Boynton et al. 2017). It should be noted that differences (or changes) in the composition of the GM, typically assessed via 16S rRNA amplicon sequencing, do not necessitate differences (or changes) in the function of those bacterial communities or model phenotypes. Clearly, these factors must be considered in the context of which are most likely to change inadvertently, or go unrecognized by researchers or unreported in the literature, and thus serve as confounds or sources of poor reproducibility between studies. For example, factors such as high-fat diet are recognized to strongly influence the GM and host physiology, and as such, are unlikely to go unrecognized as an explanation for discrepant findings. In contrast, the difference between standard maintenance and breeder chows are less pronounced, and both types of chow might be kept in the same room of a vivarium. While uncommon, examples exist of changes in rodent model phenotypes due to occult differences in the GM associated with practices at the supplier (Rohde et al. 2007; Robosky et al. 2005), prophylactic use of antibiotics by veterinary care staff (Miller et al. 2015), unknown features at different institutions (Yang et al. 2013), and other factors.

## Source of mice, whether commercial or colleague, may determine model phenotype

Considering the relative “effect size” of the aforementioned factors and evidence supporting each factor as a potential source of poor reproducibility (i.e., discordant findings between labs or over time), supplier-dependent differences are likely the best supported. The subtle environmental and procedural differences between the primary suppliers of inbred laboratory mice (Jackson, Taconic, Charles River, and Envigo in the U.S., plus Janvier in Europe, and CLEA Japan in Asia) are associated with reproducibly different GM characteristics (Ericsson et al. 2015; Wolff et al. 2020; Hilbert et al. 2017; Rasmussen et al. 2019). While the host genotype also influences the composition of the GM (Hufeldt et al. 2010; Hildebrand et al. 2013; Kovacs et al. 2011), those differences (e.g., between A/J and C57BL/6 mice from the same supplier) are typically outweighed by supplier-dependent differences (e.g., between C57BL/6 from two different suppliers). Even within the same source of animals, it should be noted that these suppliers have multiple production facilities with mice harboring different GMs (Ericsson et al. 2015). Similarly, the GM in rodents from the same production facility, and even the same isolator within that facility, can be expected to change subtly (or unexpectedly) over time, reflecting the dynamic nature of the GM on a population level (Mandal et al. 2020). While it’s impossible to control for subtle changes in the production colony over time, many investigators will request animals from a specific isolator when ordering mice. At the initiation of a new production colony of inbred mice, most producers begin with rederivation via embryo transfer (ET) of the desired strain into pseudopregnant surrogate dams (typically a hearty outbred stock) colonized with the Altered Schaedler Flora (ASF) (Dewhirst et al. 1999). Following parturition, the pups are colonized with the eight defined and culturable bacteria contained in ASF, and allowed to breed and grow the colony to production-level capacity. During this period, mice are often maintained in open-top cages and the acquisition of the additional bacteria detected in the GM of an inbred mouse occurs somewhat stochastically, slowly gaining richness and diversity over multiple generations, in a purely passive fashion with no intentional inoculation of most “over-the counter” inbred strains. Indeed, several members of the ASF can be identified in fecal samples of inbred mice from most suppliers annotated as *Mucispirillum schaedleri* (ASF 457), *Ruminococcus gnavus* (ASF 502), *Parabacteroides distasonis* (ASF 519), and *Lactobacillus murinus* (ASF 361). The MU MMRRC has maintained up to four separate colonies of CD-1 mice, each colonized with a microbiome originally derived from a different commercial supplier, initiated via embryo transfer (ET) of CD-1 embryos into C57BL/6 mice purchased from each supplier and carried to term (Hart et al. 2018). The resulting offspring have been maintained minimally inbred via rotational breeding and periodic introduction (again via ET) of new genetic stock. While the GM has remained relatively stable in these colonies over 40 + generations, we have noted subtle shifts following room changes (over multiple generations) or transfer to other institutions (Hart et al. 2018), reflecting its considerable, but not absolute, resiliency to subtle environmental pressures. It is worth noting that, when considered collectively, such institution-specific effects on the GM can occur both acutely and over extended periods of time and multiple generations. The GM of these CD-1 colonies in our institution are rigorously monitored, using a quarterly colony survey based on 16S rRNA sequencing, followed by comparison to data from the previous quarter, as well as earlier historical data. While β-diversity does not change to any detectable degree from generation to generation, careful scrutiny of richness has demonstrated slow and subtle transgenerational decreases in richness within GM4, the richest of these semi-standardized GMs, potentially indicating a very gradual institutional effect requiring many generations to manifest. To counteract these processes, new C57BL/6 mice are purchased from each supplier annually and used as surrogate dams for rederivation and introduction of fresh CD-1 genetics into the colony. In this manner, both the host genetics, and colony GM are periodically ‘refreshed’, to maintain genetic heterozygosity and distinct differences between GM profiles, respectively.

Studies of GF mice have identified an extensive list of GM-mediated developmental influences. In addition to the induction of mucosa-associated lymphoid tissue (MALT) such as isolated lymphoid follicles (ILFs), stimulation of host pattern recognition receptors (e.g., Toll-like receptors, Nod-like receptors, lectins) by microbe-associated molecular patterns (MAMPs) induces increased vascularization, crypt elongation and villous remodeling, and epithelial production of antimicrobial peptides [reviewed here (Chinen and Rudensky 2012; Hooper et al. 2012; Cebra 1999)]. Of note, ASF-colonized mice demonstrate a phenotype intermediate between GF and SPF mice in terms of maturation of the immune system (Bleich and Hansen 2012), suggesting that the relationship between the GM richness and multiple processes of immune system development exists on a gradient. Moreover, there is variability *within* SPF mice with regard to both richness and immune system development, and both measures can be pushed even further through the use of wild-caught or pet store mice (Beura et al. 2016). Interestingly, ‘humanized’ mice harboring a human GM display an adaptive immune system closer to that of ASF-colonized mice than standard SPF mice (Chung et al. 2012), demonstrating the co-evolutionary relationship between the GM and host species. This is *not* to say however that humanized mice are inherently flawed experimental models, as their advantages are discussed below.

Similarly, the influence of the GM on neurodevelopment and adult behavior is apparent in comparisons between GF and SPF mice, but also between different SPF microbiota. Behavioral studies have demonstrated a wide range of neurodevelopmental differences in GF and antibiotic-treated mice, including abnormal social interaction (Desbonnet et al. 2014) and response to stress (Sudo et al. 2004) as well as altered neurogenesis and gene transcription in the brain (Wang et al. 2020; Ogbonnaya et al. 2015; Stilling et al. 2015). While differential neurodevelopment and behavior in the complete absence of microbes is noteworthy, other studies have shown that differences in the composition of complex SPF microbiota are causally associated with behavioral differences, and experimental transfer of the GM can either mitigate or exacerbate anxiety-related behaviors in mice (Collins et al. 2013). Elegant studies from the Cryan lab have demonstrated a role for vagal communication in these afferent CNS signals from the gut (Bravo et al. 2011). The fact that naturally occurring strain-dependent GMs can causally induce changes in anxiety-related behavior, suggests that supplier-dependent differences (which typically outweigh most strain-dependent compositional differences) could also exert such effects.

These source-dependent differences, serving as potential contributors to poor reproducibility in mouse models, can often be attributed to specific bacterial taxa. One classic example of a supplier GM-dependent influence on the host phenotype led to the identification of segmented filamentous bacteria (SFB) as critical and potent inducers of mucosal immunity and colonization resistance against pathogens (Ivanov et al. 2009; Talham et al. 1999; Umesaki et al. 1999). These studies also exemplify the conversion of poor reproducibility into a series of monumental discoveries; while SFB had been visualized on microscopy, adherent to the ileal mucosa of a multitude of host species for decades, it wasn’t until those crucial observations of differential IL-17 production in mice from two different commercial suppliers, that SFB were identified as keystone species, not just in mice, but likely in humans as well (Chen et al. 2018; Yin et al. 2013). Quickly following suit, several other inbred mouse models of immune-mediated disease driven by CD4^+^ Th17 cells reported enhanced disease severity in association with SFB colonization (Lee et al. 2014; Lee et al. 2011; Wu et al. 2010; Stepankova et al. 2007), while Th1-dependent models actually reported decreased disease severity (Kriegel et al. 2011). How many discordant findings in studies of Th-17-mediated autoimmune diseases, performed prior to Ivanov and Littman’s identification of SFB’s role in immune system development, could be explained by the presence or absence of SFB?

Similarly, *Helicobacter* spp. remains a prevalent colonizer of research mice in facilities worldwide (Shames et al. 1995; Taylor et al. 2007; Duangchanchot et al. 2014), despite being on the exclusion list of most suppliers. The influence of *Helicobacter hepaticus* on model reproducibility was first highlighted through its role as a cause of chronic active hepatitis and liver cancer in toxicology studies performed at the National Center Institute in the early 90s (Ward et al. 1994; Fox et al. 1994), followed by the identification of a second enterohepatic strain, *H. bilis* (Fox et al. 1995). These findings spurred research activity on the influence of this bacterium on host immunity, and it was soon appreciated that a vast number of mouse models of inflammatory bowel disease and colorectal cancer were largely dependent on the colonization of mice with one of these *Helicobacter* spp., reviewed in depth elsewhere (Foltz et al. 1998; Fox et al. 2011). While induced or enhanced via colonization with *Helicobacter* sp., these models nonetheless rely on a genetically susceptible host, as colonization with these *Helicobacter* spp. is clinically silent in most inbred mouse strains. Of note however, many of these models are also dependent on the presence of a background microbiota, as mice mono-associated with *H. hepaticus* often fail to develop disease (Sellon et al. 1998; Nagalingam et al. 2013; Dieleman et al. 2000). This may be explained by studies in ASF-colonized mice demonstrating heterologous mucosal immune responses induced by *H. bilis* (Jergens et al. 2006, 2007). Thus, Helicobacters are often considered provocateurs of mucosal immune responses against the background microbiome, in genetically susceptible hosts. In contrast to the non-specific Th17 immune responses induced by SFB, *Helicobacter* spp. are historically associated with Th1 immune responses (Whary et al. 1998). Interestingly, like many other *Proteobacteria* (Ivanov et al. 2009; Garland et al. 1982; Heczko et al. 2000), colonization of *H. hepaticus* may also depend on the presence or absence of SFB (Wolfe et al. 2020), and supplier-dependent differences in the abundance of *Enterobacteriaceae* are also associated with differential colonization resistance against Salmonella (Velazquez et al. 2019). Thus, models of infectious disease, as well as models that are induced via experimental inoculation with live bacteria, are potentially (if not likely) susceptible to supplier-dependent microbial influences, and there is the potential for interactions between resident and experimentally administered taxa.

**Fig. 1:**
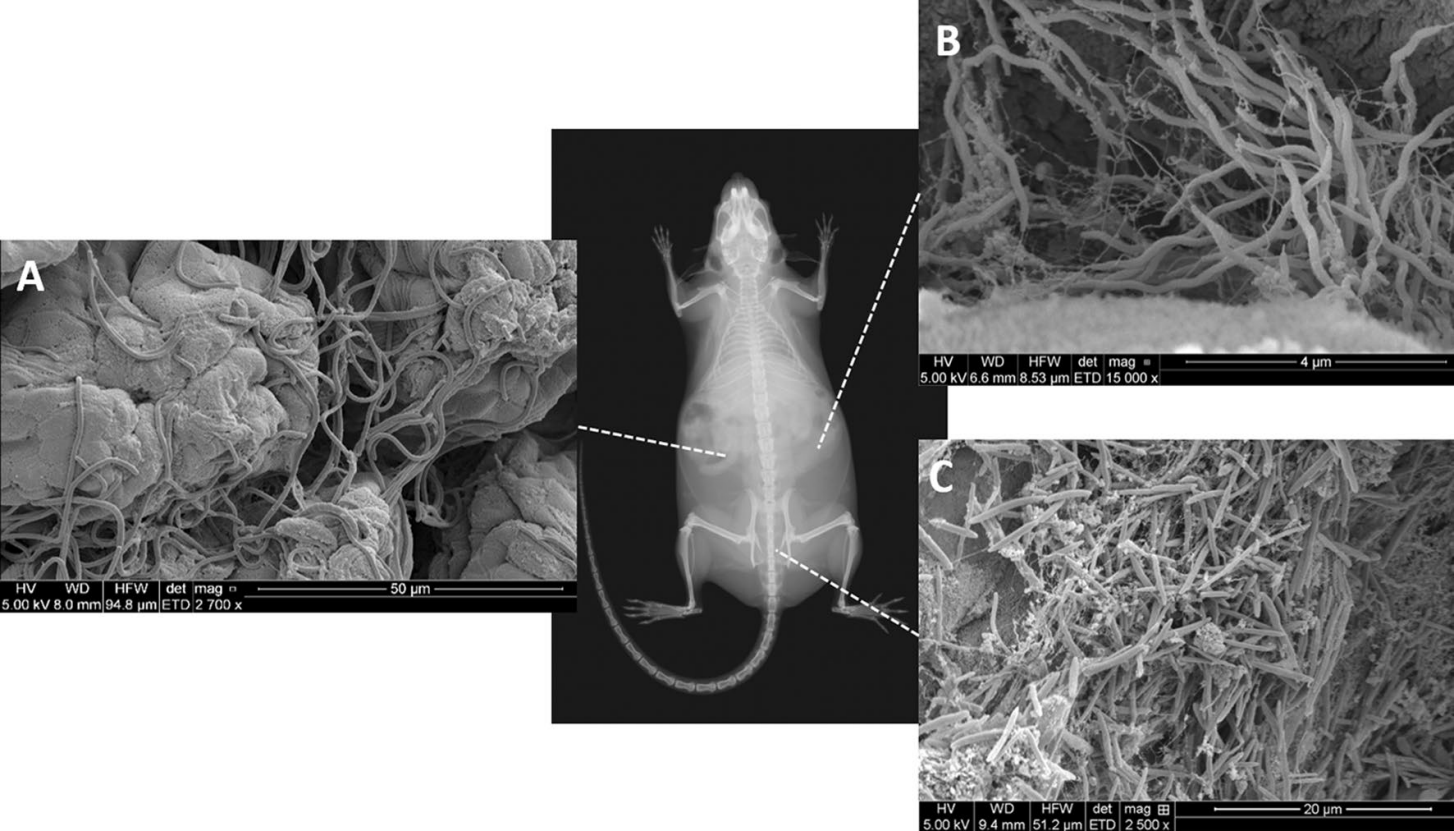
Scanning electron microscopy (SEM) images of several taxa capable of influencing host immune responses and physiology at different regions of the gut, including (**a**) segmented filamentous bacteria adherent to the ileal mucosa, (**b**) *Helicobacter hepaticus* within a cecal mucosal fold, and (**c**) a mixed bacterial community in the colon containing many different taxa, including *Akkermansia* spp. (not visualized)

Other resident gut bacteria of interest include *Akkermansia* spp., and *Lactobacillus* spp. As the type strain for the order *Verrucomicrobiales*, *Akkermansia muciniphila* has gained attention for several reports of its association with increased insulin sensitivity, positive glucose regulation, and possibly an adjunct mechanism through which the anti-hyperglycemic agent metformin may act (Shin et al. 2014; Cuesta-Zuluaga et al. 2017; Rosario et al. 2018; Lee et al. 2018). It is a common member of the fecal microbiota in mice from many sources and is cultivable and available through the ATCC (BAA-835 and BAA-2869), lending itself to controlled experimentation. As it is frequently not affected by antibiotics (Korte et al. 2020), it may proliferate in the context of antibiotic pressures and exert an increased effect on the host phenotype (Hansen et al. 2012). In addition to *L. murinus* and *L. intestinalis* (presumably of ASF origin), several other *Lactobacillus* spp. are found in the GM of inbred mice, including *L. reuteri*, *L. gasseri*, and several other unresolved strains. Changes or differences in the relative abundance of Lactobacillus are noteworthy for several reasons. There is a growing body of evidence that several endogenous species of *Lactobacillus* spp. function as ‘psychobiotics’, or live bacteria with anxiolytic effects on the host (Bravo et al. 2011; Liu et al. 2016; Sarkar et al. 2016; Reis et al. 2018). Like Akkermansia, Lactobacillus are often spared during certain antibiotic regimens and could ostensibly affect host physiology or behavior in such scenarios. As with SFB, colonization by *Lactobacillus* spp. may be challenging to accurately quantitate *ante-mortem* due to its dominance in the upper GI tract and relatively minor presence in fecal microbiota (Fig. 1).

However, these taxa represent but a handful of genera, and while there appears to be a degree of consistency in the influence of these bacteria on the induction of specific components of the immune system and downstream phenotypic changes in models driven by those arms of the immune system, the influence of the entire complex bacterial community present in mice from different commercial suppliers is less clear. In models driven by the same genetic predisposition, the effects appear to be consistent as in IL-10-deficient mice on either a C57BL/6 or C3H/HeJ genetic background. In this GM-dependent model (Yang et al. 2013; Sellon et al. 1998), while the host genetics also profoundly influence the composition of the mature GM, mice colonized with GM derived from Jackson and Taconic mice experience significantly greater disease than mice colonized with the GM derived from Charles River mice, regardless of the background genetics (Hart et al. 2017).

Similarly, in experiments with isogenic mice, Jackson-origin GM is associated with more severe disease in DSS-induced colitis when compared to Envigo-origin GM (manuscript in preparation). Considering the inverse relationship between the GM richness and disease severity in these murine models of IBD, it is tempting to note the similar observations made in human IBD populations. In contrast however, the *Apc*^+*/min*^ model of colorectal cancer is actually protected by the low richness Jackson GM relative to the rich Envigo GM (Moskowitz et al. 2019). Collectively, this suggests that the different supplier-dependent GMs do not universally exacerbate or ameliorate intestinal disease, but rather exert model-specific influences, depending on the disease mechanism.

Scenarios in which a different supplier-dependent GM may unknowingly be introduced vary. Transient exposure to mice harboring a different microbiota (or their fecal material), can lead to persistent changes due to coprophagy. As an example, following timed mating of two mice with different supplier-dependent GMs wherein the dam and sire are together for only three days, sufficient time is spent together that the GM of the resulting offspring will likely reflect that of both the dam and sire. Other examples include the sharing of resources (i.e., mice) between labs, where the detailed exposure history of mice may be unknown.

## Diet and other important considerations in model reproducibility

The influence of diet on the GM of inbred mice, and thus reproducibility between studies, depends on the degree of change or difference in macromolecular content. For example, the significant effects of a high-fat diet on the composition of the GM have been demonstrated many times (Bolnick et al. 2014; Carmody et al. 2015) and mirror those observed in humans (David et al. 2014). Org et al. (2015) performed an innovative series of cross-foster and GWAS studies using mice from the Hybrid Mouse Diversity Panel (HMDP) (Bennett et al. 2010) to demonstrate GM control of dietary response to a high-fat, high-sucrose diet, as well as specific heritability and putative loci for particular gut bacteria. However, these types of diets are typically used as experimental variables expected to induce substantial changes in the GM, and are unlikely sources of poor reproducibility. In contrast, differences or changes between commercial chow formulations of comparable macromolecular content induce either extremely subtle (Ericsson et al. 2018) or transient (Ma et al. 2012) changes in the GM. However, given the significant sex × diet interactions in the influence of high-fat diets on the GM of inbred mice (Org et al. 2016; Bolnick et al. 2014), it is possible that strains other than those reported in the literature (FVB and outbred CD-1) may react differently to a change in commercial source of comparable chow. Supporting that notion, many of the studies referenced above demonstrate significant interactions, wherein the effect of one factor is dependent on another factor. Similarly, we have detected robust interactions between bedding and caging type (Ericsson et al. 2018), and between bedding and method of water disinfection (Bidot et al. 2018). Interestingly, these studies also revealed that dramatic differences in the small intestinal or even cecal bacterial communities associated with these husbandry variables are often undetectable in the feces. Thus, even the short colonic transit time can mute upstream effects that may or may not lead to phenotypic differences. This raises the question of how much the GM present in the upper gastrointestinal tract (GIT) affects host phenotype.

Stronger environmental pressures have also been shown to exhibit differential effects on the gut microbiota, depending on its composition. For example, C57BL/6 mice harboring the GM of a wild mouse are extremely resistant to antibiotic-induced changes when compared to traditional SPF mice with either a Jackson or Taconic-origin GM (Rosshart et al. 2019) (both comparably sparse SPF microbiomes). In contrast, the relatively low and high richness GM observed in mice from Jackson and Envigo appear to respond very similarly to several commonly used antibiotics (Korte et al. 2020). While it might not be intuitive that antibiotic exposure would go unnoticed, or at least unappreciated, scenarios for such influences include exposure to tetracycline (Yin et al. 2015) or doxycycline (Boynton et al. 2017) to induce or silence gene expression, or the use of topical triple antibiotic ointment by veterinary care staff to treat fight wounds or dermatitis, which is subsequently ingested during grooming (Korte et al. 2020). It should also be noted that, despite a lack of appreciable effect on the GM as characterized using 16S rRNA amplicon sequencing, antibiotics may nonetheless affect model phenotypes via GM-independent mechanisms or changes in the GM below the resolution of current methods. As an example, chronic exposure to trimethoprim-sulfamethoxazole (TMS) results in no more change in the GM over time than is observed in untreated control mice, whether mice are colonized with a sparse Jackson-origin GM or a rich Envigo-origin GM. Yet, TNFR-deficient 2D2 TCR-transgenic mice, with an incredibly robust, sex-biased neurodevelopmental phenotype modeling Devic’s disease, demonstrated a complete loss of phenotype following colony-wide administration of TMS (using the same dosage and route of administration) in response to poor breeding performance and a suspected occult bacterial etiology. Supporting a GM-mediated role in the loss of phenotype, co-housing of mice with wild-type mice purchased from the original source resulted in a complete recovery of the phenotype, and novel insights into the disease pathogenesis (Miller et al. 2015). Scenarios such as this underscore the value of a thorough knowledge of the GM of origin in the strains being used, as well as periodic banking of fecal samples in a − 80 °C freezer, to provide a historical record of the GM in each colony, as well as a possible source for re-inoculation should the need arise.

## Model phenotypes lost in translation

Frequently, even robust and reproducible experimental results generated in mouse models cannot be replicated in other model species, or humans. If an investigational new drug (IND) potently inhibits tumor growth in multiple mouse models of hepatocellular carcinoma (HCC), but has no efficacy whatsoever in canine or human HCC patients, it has little hope of testing beyond Phase I clinical trials, much less reaching the market. An improved ability to screen out those ‘false positives’ that fail to produce comparable benefit to humans, or even worse, are actually detrimental to humans, would increase efficiencies in the drug development pipeline by eliminating false leads. Equally important, an enhanced ability to identify ‘false negatives’, i.e., those INDs that failed to show efficacy in animal models but that might have efficacy in humans, would be expected to increase the number of candidate compounds in the pipeline. Several lines of research now suggest that the GM of research mice can be manipulated so as to enhance the translatablity of research using those mice, to the human condition.

A rather prescient opinion piece by Pedersen and Babayan in 2011 (Pedersen and Babayan 2011) posited that studies performed in wild animals might provide more meaningful outcomes in translational research, owing to their outbred nature, history of repeated antigen exposure, and primed immune system (Boysen et al. 2011). Five years later, the work of Stephen Jameson and David Masopust at the University of Minnesota (Beura et al. 2016; Huggins et al. 2019), Herbert “Skip” Virgin at Washington University (Reese et al. 2016), and Stephan Rosshart and Barbara Rehermann at the NIH (Rosshart et al. 2017, 2019) would resoundingly support this proposal, based on studies performed using various populations of wild-caught and pet store-origin mice. Ongoing work in those and other labs, including the MU MMRRC, often in conjunction with commercial suppliers, continue to investigate the biological effects of a wild mouse GM (reviewed in detail by J-K Seong in the present issue), and develop and refine animal models through customized GM colonization and antigen exposure. One of the greatest impediments in the wide-spread adoption of these approaches is the history that has created our current research environment and practices. Pioneers in the development of the first inbred mouse lines recognized early on the influence of not just host genetics, but also infectious agents, on experimental reproducibility. Beginning with those pioneers, and extending to the present-day distribution of dozens of different inbred strains through centralized suppliers, efforts have slowly expanded to ensure that mice are free of an ever-growing list of ‘excluded pathogens’. Animal care staff at these centralized supplies are typically under strict guidelines regarding their exposure to pet and wild rodents, lest they serve as a disease vector and transmit something to their facility. While the above practices follow a certain logic, they result in laboratory mice with negligible antigenic stimulation during early life. This, along with the practices employed to seed the GM of founders in a new production colony, have also resulted in a substantially decreased GM richness regardless of commercial source and the exclusion of recognized immunostimulatory bacteria such as *Helicobacter* spp. that are endemic in wild populations. Thus, the historical desire to eliminate and exclude any and all overt and opportunistic pathogens from all research animals (ostensibly enhancing reproducibility at the expense of translatability) is confronted with mounting evidence that the traditional SPF paradigm may be insufficient or even inappropriate for some studies. The decision as to whether or not wild mice, or mice intentionally infected with subclinical immunostimulatory agents like *Helicobacter* spp. and MNV, would be allowed in an institutional vivarium has multiple stake-holders including other researchers in the facility and the attending veterinarian, and such decisions require careful consideration. While controlled experimental inoculation with known agents has the advantage of reliance on a limited number of known inoculants with known transmission routes and available methods of screening, the pathogen burden and commensal GM richness of pet store or wild mice is vastly greater and thus likely of greater concern to others in the same vivarium, and more labor-intensive to survey a facility. However, the distinct advantage to working with wild mice or ‘wildling’ inbred mice, is the strong phenotype induced by those antigenic exposures during development, and the improved predictive power with regard to human outcomes. Ideally, the use of mice with increased antigen exposure would represent an adjunct, rather than replacement, of current SPF mouse models, as discrepant phenotypes between otherwise isogenic mice harboring distinct GM or antigen exposure histories would suggest a GM-mediated influence on the physiologic, or pathologic, process under investigation, and rationale for continued investigation.

## Transforming poor reproducibility into discovery

While many studies are intentionally, and prospectively, designed to make new discoveries related to the microbiome and its influence on some aspect of host physiology, other studies are borne of unexpected results or discrepant data between studies or labs, and the curiosity of an investigator. Examples abound in the literature, particularly in the context of notoriously “fickle” models with variable phenotypes. A classic example is the non-obese diabetic (NOD) mouse model of autoimmune (type I) diabetes mellitus.

Reports of opposing effects of acidified water on the GM and disease phenotype (Wolf et al. 2014; Sofi et al. 2014) were recognized as an opportunity, and pursued by Zhao and Tarbell to reveal a lack of effect in either direction (Zhao et al. 2014). Collectively, the findings from these studies suggest that other variables likely influenced the model phenotype, providing fertile ground for continued investigations of the microbial taxa or functions that are mechanistically involved in the different disease presentations. Similarly, different effects of treatment with vancomycin have been reported in NOD mice, with initial reports indicating that early life exposure to the selective Gram-positive antibiotic resulted in reduced diabetes incidence (Hansen et al. 2012), findings of particular interest in the context of earlier studies demonstrating similar or even exacerbated disease incidence in GF NOD mice (Alam et al. 2011; King and Sarvetnick 2011). Subsequent studies however, produced seemingly conflicting results, with vancomycin administration leading to increased disease incidence (Hu et al. 2016; Brown et al. 2016; Candon et al. 2015). As with acidified water, the different findings likely reflect differences in the baseline GM of mice used in each study, or a host of other genetic or environmental factors interacting with the variable of interest. These are but two examples (in only one model) of discrepant findings seeding the next line of investigation. In this context, lack of reproducibility between studies should be viewed as a challenge to identify the factors responsible for the discordant findings, rather than dismissed as problematic events resulting from poor experimental design or inappropriate statistical analyses.

## Best practices at all levels of science

The considerations described above present both challenges and opportunities, for individuals working at almost every level of biomedical research. For the suppliers producing commercially available laboratory mice, there is a growing burden to recognize and report the characteristics of the GM in their colonies. Specifically, a production colony surveyed via 16S rRNA amplicon sequencing could be reported online, and would cost little more than the battery of diagnostic tests performed on sentinel mice. As with any other physiological parameters provided by the supplier, these data add value to the end users. Similarly, health reports would ideally be expanded to include important commensal players such as SFB and *Akkermansia* sp., allowing researchers to make informed purchases.

Repositories and other federally funded distributors of research mice should similarly be cognizant of the GM in their colonies, particularly that of surrogate dams used as recipients in embryo transfer procedures used to resuscitate frozen germplasm. The CD-1 colonies maintained at the MU MMRRC are available for that very purpose, allowing ‘customization’ of a research model via rederivation using surrogate dams harboring the GM of interest, or rederivation of germplasm in dams from more than one colony to prospectively assess the influence of the different GMs.

Scientists are encouraged to be aware of the factors discussed above, and to be forward-thinking with regard to the mouse models being used in their research. At the most basic level, this might constitute something as simple as periodically banking fecal samples, while more proactive measures might include efforts to optimize the phenotype of mouse models through intentional colonization with the GM of other suppliers, or even a wild mouse GM. Methods of identifying or validating an association between certain environmental/dietary features, the GM, and changes or differences in a phenotypic outcome are many and varied. In most cases, demonstration of causal relationships will require manipulation of the GM, including complete or partial ablation of the GM, supplementation with specific taxa, or exposure to complex GM. While a detailed discussion of available approaches is beyond the scope of the current review, readers are directed to the following literature (Ericsson and Franklin 2015; Lundberg 2019; Lundberg et al. 2016; Ericsson et al. 2017a) describing various GM-related experimental design considerations. It should be remembered that the actual transfer of the GM itself (rather than the composition of the GM being transferred) can influence the host, and procedural controls are required. For the same reasons, regardless of the transfer methods used, studies should be performed or repeated in second generation mice born to the actual GM recipient mice as the latter are naturally colonized by multiple sources of microbiota (i.e., vaginal, fecal and cutaneous) beginning at birth. Additionally, the success of GM transfer in methods such as co-housing and even repeated gastric gavage following antibiotic exposure, is largely dependent on the difference between donor and recipient in starting richness (Ericsson et al. 2017b). Other ‘best practices’ include the careful consideration of cage density in the experimental design. Due to coprophagy, mice within a cage are frequently more similar to cagemates than to littermates in other cages. This is particularly evident after application of a selective pressure such as antibiotics. Group-housing has obvious benefits with regard to animal welfare and reduced housing costs, but in the event of pronounced ‘cage effects’, it becomes problematic to consider individual mice as the biological unit. In certain scenarios, such as determining the influence of a specific host genotype on the GM, co-housing can serve as a valuable confirmation of genotype-dependent effects. For example, a significant difference in GM composition between WT, heterozygous, and KO littermates is substantially strengthened if these differences are maintained in co-housed mice of different genotypes (Bains et al. 2019). If the goals of the experimental design are to normalize the GM among all groups, rather than validate genotype-dependent differences, co-housing may be insufficient as mucosal communities may remain distinct (Robertson et al. 2019).

Regarding sample collection, one of the primary confounds to consistent data is the time of day that samples are collected. Being nocturnal, mice are typically active during the dark cycle, and fecal throughput is much more rapid and frequent than during the light cycle. Accordingly, the composition of fecal samples from a group of mice collected 12 h apart will differ substantially (Kuang et al. 2019; Thaiss et al. 2016). As samples are best collected fresh and then processed or frozen quickly, we recommend collecting early in the morning as the dark cycle has just ended. There are numerous other best practices related to sample processing, library preparation and sequencing, and the bioinformatics tools used to filter and annotate the data that are beyond the scope of this review. Readers are referred to excellent reviews by Pollock et al. (2018) and Knight et al. (Knight et al. 2018) for these details.

Ultimately, faced with changes or loss of a model phenotype, researchers are encouraged to view the situation as an opportunity for discovery, and pilot studies to identify the cause and mechanism underlying the change are warranted. Our understanding of gene function has been elucidated primarily through loss- and gain-of function approaches using mouse models, and similar strategies can be applied to better understand the influence of the GM on model phenotypes, and host physiology.
